# A T-cell independent universal cellular therapy strategy through antigen depletion

**DOI:** 10.7150/thno.66832

**Published:** 2022-01-01

**Authors:** Dan Li, Wenbing Wang, Shufeng Xie, Maolin Ge, Ruiheng Wang, Qiongyu Xu, Yan Sun, Jiang Zhu, Han Liu

**Affiliations:** Shanghai Institute of Hematology, State Key Laboratory of Medical Genomics, National Research Center for Translational Medicine at Shanghai, Rui Jin Hospital, School of Medicine and School of Life Sciences and Biotechnology, Shanghai Jiao Tong University, Shanghai, China.

**Keywords:** cellular therapy, CD19, CAR-T, cytokine release syndrome, artificial antigen-recognizing cell

## Abstract

**Rationale:** T cell therapeutic strategy using CD19-targeting chimeric antigen receptor (CAR) is a revolutionary, novel, and successful treatment for B-cell malignancies. However, the dependency on T-cell mediated cytotoxicity restricts CAR-T therapy as a patient-specific individualized therapy with severe side effects, such as cytokine release syndrome (CRS). Whether a non-T-cell based universal cellular therapy can substitute CAR-T therapy is largely unknown.

**Methods:** Various artificial antigen-recognizing cells were prepared to determine whether non-T-cell-derived CD19-scFv bearing effector cells could cause target cell death. A universal strategy for CRS-free cellular therapeutics was proposed, utilizing artificial antigen-recognizing cells (AARC), which can be manufactured universally and routinely as “off-the-shelf” mesenchymal stromal cells (MSCs) or other types of non-autologous cells expressing anergic CARs.

**Results:** We demonstrated that T-lymphocytic and non-lymphocytic cells could cause CD19 internalization and subsequent depletion when armed with a CD19-recognizing moiety. This CD19 antigen depletion could efficiently induce T-cell independent apoptosis in target cancer cells whose survival depends on CD19 expression, suggesting that CD19 antigen depletion constitutes a crucial tumor destroying mechanism for CD19-CAR-T, especially for its long-term efficacy.

**Conclusion:** Our results uncovered an unrecognized CAR-T cytotoxicity and antigen loss mechanism and provided new insights into a shift from unique patient-specific autologous therapeutics to universal and standardized allogeneic treatment.

## Introduction

Autologous CAR-T cells constitute a promising novel therapeutic approach for the treatment of hematological malignancies. Although this individualized therapy-based approach has resulted in outstanding clinical results to date, it has several intrinsic disadvantages, such as the risk of manufacturing failure in certain patients, the risk of delay in treatment due to time-costing manufacturing procedures, as well as financial burdens. Therefore, developing the next-generation allogeneic cellular therapeutic strategy to address these issues is an active area of research 1.

Cytotoxic T lymphocytes (CTL) mediate their anti-tumor effects predominantly through the granule exocytosis axis, the death ligands axis, and the release of cytokines 2. As one of the main adoptive T-cell therapy (ACT) approaches, CAR-T cells also mediate target cell death mainly through these pathways 3. Most adoptive studies have focused on the CD8+ T-cells because of their direct cytolytic activity. However, preclinical and clinical data have established the importance of incorporating CD4+ T-helper cells during immunotherapy 4. Moreover, the adoptive transfer of CD4+ T-cell populations has shown that these cells can cause tumor regression 5. Interestingly, although CD4+ CAR-T cells are slower than CD8+ CAR-T cells for instigating target cell death, the single-cell analysis revealed that these cells achieved the same degree of tumor cell death 6. These results suggested that the direct cytolytic activity may not be absolutely required for CAR-T cell mediated target cell death. It has been proposed that CD4+ T-helper cells can aid the survival of infused CD8+ T cells, induce tumor cell senescence through derived cytokines, and even differentiate into cytolytic effectors 7-9. Nevertheless, whether T-cell independent mechanisms are involved in anti-tumor effects has not yet been addressed.

Successful CAR-T therapeutics depends heavily upon the *in vivo* activation and expansion of CAR-T-cell products, which inevitably cause the well-characterized side effect of cytokine release syndrome (CRS). Therefore, the development of T-cell independent universal cellular therapy strategies may provide an alternative option for “off-the-shelf” and standardized treatments and reduce the CRS risk. Here, we have demonstrated that CAR-T cells can destroy target cells through a T-cell independent mechanism. Based on this finding, we propose the concept of a universal cellular therapy strategy, which could be used in conjunction with current CAR-T therapeutics.

## Methods

### Cell lines and primary cells

SEM, REH, RAJI, Jurkat, and K562 cell lines were obtained from DSMZ. KOPN8, KOBP26, and NALM6 cell lines were obtained from ATCC. Mesenchymal stem cells (MSCs) were obtained from Shanghai Nerostem Tech. CD3+ T cells were isolated using EasySep Human T Cell Isolation Kit (STEMCELL Technologies) and then cultured in CTS T Cell Expansion medium (Thermo) containing 10% fetal bovine serum and 100 IU/ml human IL-2 (PeproTech). The CellTiter 96 MTS assay (Promega) was used to determine cell viability and proliferation.

### Plasmid constructions

Fragments encoding CD19-, CD22-, and CD133-specific competent CARs and anergic CARs (scFvs) that lack co-stimulatory and ζ-chain signaling domains were inserted into the lentiviral vector pCDH-T2A-copGFP (System Biosciences). The CD19-mRuby2 fusion was generated by fusing the mRuby2 sequence at the C terminus of CD19 and cloned into the pCDH lentiviral vector. Target sequences (CTTCAACGTCTCTCAACAGAT #1 and CCGAGTTCTATGAGAACGACT#2) against human CD19 and a control scrambled sequence (CTCAATCAACAGATCTCGTCT) were inserted into the pLKO.1 vector (Sigma).

### Flow cytometry

The CellTrace Far Red Proliferation Kit, the CellTrace CFSE Cell Proliferation Kit and the CellTrace Violet Proliferation Kit (Invitrogen) were used for cell labeling. The human CD19-APC and CD69-APC antibodies were obtained from BD Biosciences and the human CD133-PE antibody was purchased from Miltenyi Biotec. A human CD22 antibody was obtained from Biolegend (San Diego, California). Apoptosis was measured using the Annexin V Apoptosis Detection Kit (BD Bioscience). Flow cytometry was performed on LSRFortessa or FACSAria sorter (BD Biosciences). Data were analyzed by the FlowJo software.

### Reagents

Bortezomib (Velcade), Sc-79, CsA, and dynago-4a were obtained from Selleck Chemicals. Bafilomycin A1 (Baf-A1), DC661, MK-2206, and MβCD were acquired from MedChemExpress.

### Immunoblots

Human CD19 and Akt antibodies were obtained from ABclonal Technology. Antibodies against CD133, p44/42 MAPK (Erk1/2), phosphor-p44/42 MAPK (p-Erk1/2), and phosphor-Akt (p-Akt) were purchased from Cell Signaling Biotechnology. MYC antibody was obtained from Santa Cruz Technology. Mouse anti-GAPDH antibody was obtained from Sigma Aldrich, and immunoblot signals were acquired by the Amersham Imager 600 (General Electric Company).

### Image flow

SEM cells labeled with Cell Trace Far Red and CD19 CAR-Jurkat T cells expressing copGFP were co-cultured at the ratio of 1:1 for 1 h. Cells were re-suspended in 4% PFA for 30 min, and images were acquired on the Amnis Imagestream Mk II Imagine flow cytometer (Luminex).

### Super-resolution imaging

REH cells expressing CD19-mRuby2 fusion labeled with Cell Trace Violet and CD19 CAR-Jurkat T cells expressing copGFP were seeded in cell culture imaging dishes. Protease inhibitor cocktail was added to prevent CD19 antigen degradation. Images were acquired on the GE Delta Vision OMX SR imaging system, and ImageJ software was used to generate the figures.

### qRT-PCR

qRT-PCR was performed using 7500 Real-Time PCR Systems (Applied Biosystems). The data represent relative mRNA levels normalized to *GAPDH*. Primers used for qRT-PCR assays are listed below:

CD19-Forward: CCCAAGGGGCCTAAGTCATTG,

CD19-Reverse: AACAGACCCGTCTCCATTACC;

GAPDH-Forward: GGCACAGTCAAGGCTGAGAATG,

GAPDH-Reverse: ATGGTGGTGAAGACGCCAGTA.

### Mouse studies and *in vivo* imaging

SEM cells simultaneously expressing GFP and luciferase have been described previously 10. NOD/SCID mice were purchased from Vital River Laboratories. 1.5 million luciferase-expressing cells were intravenously injected via tail vein into NOD/SCID mice (five in each group), which were then administered 1.5 million scFv-expressing MSCs on a twice-weekly schedule beginning 3 days after xenograft. Total body bioluminescence was quantified at indicated time points. All animal work was performed in accordance with a protocol approved by the Animal Studies Committee of Ruijin Hospital.

### Statistical analysis

All statistical analyses were performed using the GraphPad Prism 6 software. The Student's *t*-test was used to analyze the differences between the groups.

## Results

### Target cell death through a T-cell independent mechanism

Since cytotoxic CTL- or CAR-T cells mediated target cell destruction is an acute process, the *in vitro* capability of CTLs or CAR-T cells is usually tested using a short time-specific cell lysis assay. Whether these effector cells have any chronic effects on target cells when co-cultured for an extended time has received scant attention to date.

**Figure 1:**
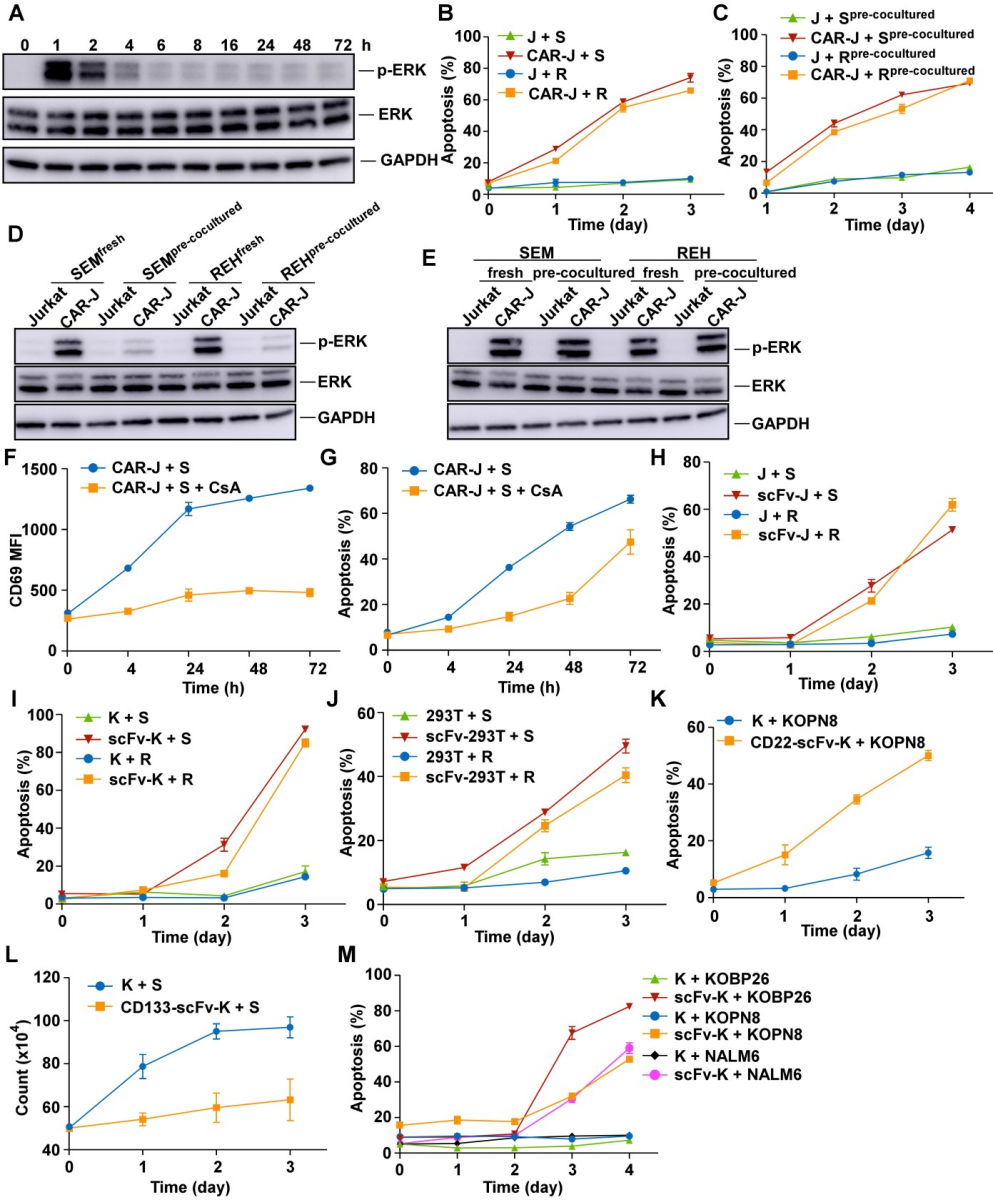
** Target cell death through a T-cell independent mechanism.** (A) Phosphorylated ERK levels of CD19 CAR-transduced Jurkat T (CAR-J) cells were determined at indicated times after co-culturing with SEM cells. (B) Specific lysis of CellTrace PE-labeled target cells was assayed by Annexin V staining at indicated times. J, Jurkat; S, SEM; R, REH. (C) Effector cells were co-cultured with target cells and separately collected by FACS sorting (pre-cocultured). The pre-cocultured target cells were re-cocultured with fresh effector cells and the specific lysis of the target cells was analyzed by Annexin V staining. (D) CD19-CAR-Jurkat or control Jurkat cells were co-cultured with fresh or pre-cocultured target cells for 1 h, and phosphorylated ERK levels in effector cells were evaluated by immunoblotting. (E) Fresh or pre-cocultured CAR-J or control Jurkat T cells were co-cultured with the indicated fresh target cells for 1 h, and phosphorylated ERK levels in the effector cells were evaluated by immunoblotting. (F) CD19-CAR-J cells were co-cultured with SEM cells with or without 200 nM CsA, and the mean fluorescence intensity (MFI) of the activation marker CD69 was evaluated by flow cytometry. (G) CD19-CAR-J cells were co-cultured with SEM cells with or without 200 nM CsA, and Annexin V staining of SEM cells was performed at indicated times. (H-J) CD19-scFv-expressing Jurkat (scFv-J), CD19-scFv-K562 (scFv-K), or CD19-scFv-293 (scFv-293) cells were co-cultured with CellTrace Far Red-labeled SEM or REH cells at the E/T ratio of 1:1. Apoptosis of the target cells was analyzed by Annexin V staining. scFv-transduced cells denote CAR-T cells lacking co-stimulatory and CD3 zeta-chain signaling domains. (K) Annexin V staining of Kopn8 cells after co-cultured with CD22-scFv-K562 or control K562 cells at 1:1 ratio. (L) CD133-scFv-K562 or control K562 cells were co-cultured with SEM cells at 1:1 ratio. Cell count of SEM cells was examined at indicated times. (M) Apoptosis of indicated target cells after co-culturing with CD19-scFv-K562 or control K562 cells. Error bars reflect ± SEM.

In a co-culture system, B cell acute lymphoblastic leukemia (B-ALL) target cells caused quick and robust activation of CD19-CAR-Jurkat T cells that was substantially reduced after 24 h as demonstrated by attenuation in ERK phosphorylation (Figure 1A and S1A). However, despite the diminished activation of effector cells, apoptosis in target cells continued to increase even after 24 h (Figure 1B). To address whether the sustainable cell death at the late time point resulted from the transient activation of T cells at an earlier time point, we collected target cells and effector cells separately after co-culturing for 24 h then cultured with fresh effector cells or target cells, respectively. Interestingly, apoptosis continuously increased in the pre-co-cultured target cells when re-co-cultured with fresh CD19-CAR-Jurkat T cells, even though they could no longer effectively activate the effector cells (Figure 1C-D). Importantly, the pre-co-cultured target cells showed only minimal apoptosis when re-co-cultured with fresh control Jurkat T cells, thus excluding the possibility that the T-cell activation mediated cytolytic activity seen in early periods was sufficient to cause the sustainable increase in apoptosis in the late period. These results suggested that target cell death might not be directly associated with T cell activation, especially in the late period. Furthermore, freshly-added target cells could still activate the pre-co-cultured CD19 CAR-Jurkat T cells (Figure 1E). This observation suggested that the diminished activation of effector cells in the late period was caused because the pre-co-cultured target cells could not activate the effector cells and not because the pre-co-cultured effector cells became non-responsive to target cells.

We suppressed CAR -T cell activation using the calcineurin inhibitor cyclosporin (CsA) to understand whether T cell activation was required for CAR T cell-mediated target cell death and tested whether target cells could still be destroyed by these anergic CAR-T cells. As expected, while CsA continuously blocked the activation of CAR T cells, as evidenced by diminished CD69 expression (Figure 1F), a large fraction of B-ALL cells still died when co-cultured for an extended time (Figure 1G).

We examined whether target cells could be killed by non-signaling CD19-CAR-Jurkat (hereafter referred to as CD19-scFv-Jurkat) cells or T cell activation was necessary for target cell death (Figure S1B). As evident from Figures 1B & H, the B-ALL target cells could undergo significant apoptosis when co-cultured with CD19-scFv-Jurkat cells, albeit the death kinetics were much slower than CD19-CAR-Jurkat cells. These results suggested mechanisms other than T-cell activation involved in the process of CD19-CAR-T mediated destruction of B-ALL cells.

Collectively, these results suggested that CAR-T cells, especially CD19-CAR-T cells, could achieve target cell death through two distinct pathways, one involving the classical CTL pathway, causing rapid death and was dependent on T-cell activation and the other with slower kinetics and was T-cell activation independent.

### Target cells are destroyed by various artificial antigen-recognizing cells

Since the activation of T cells is not crucial for target cell death, we reasoned that the target cell death effect could alternatively be achieved by other non-lymphocyte-derived antigen-recognizing cells. To this end, we determined whether non-T-cell-derived CD19-scFv bearing effector cells could cause target cell death (Figure S1B). Interestingly, the B-ALL target cells could also be killed by co-cultured CD19-scFv-K562 cells and CD19-scFv-293T cells with death kinetics similar to CD19-scFv-Jurkat cells (Figure 1I-J). These results suggested that both lymphoid and non-lymphoid cells could induce efficient target cell death when armed with an artificial antigen recognizing module.

We tested whether the targeted killing capabilities of scFv-bearing K562 cells recognizing antigens other than CD19 was a general phenomenon. Among the scFv-bearing K562 cells, CD22 targeting K562 cells could cause efficient target cell death in CD22-positive KOPN8 cells (Figure 1K). While CD133 targeting K562 cells could only retard cell proliferation without causing efficient target cell death (Figure 1L and S1C). Nevertheless, besides REH and SEM cells, other B-ALL cells such as KOPN8, KOBP26, and NALM6 cells could also be effectively killed by CD19-scFv-K562 cells, suggesting that B-ALL cells could be generally destroyed by CD19-scFv-K562 cells (Figure 1M). However, some malignant CD19-positive B cells, such as RAJI cells could not be killed by the CD19-scFv-bearing effector cells (Figure S1D-F). These results suggested that the ability of scFv-bearing effector cells to kill target cells was dependent on the target cell type and the target antigen selected. Together, these results suggested the feasibility of utilizing non-lymphocyte-derived artificial antigen-recognizing cells (AARCs) as effector cells to destroy or inhibit target cancer cells.

### Artificial antigen-recognizing cells cause antigen depletion on target cells

We next addressed the question of how AARCs can kill or inhibit target cancer cells. Since target cells can no longer activate effector CAR-T cells in the late period of co- culture, we suspected that the death or inhibition of target cells might be related to the antigen loss on target cells.

**Figure 2:**
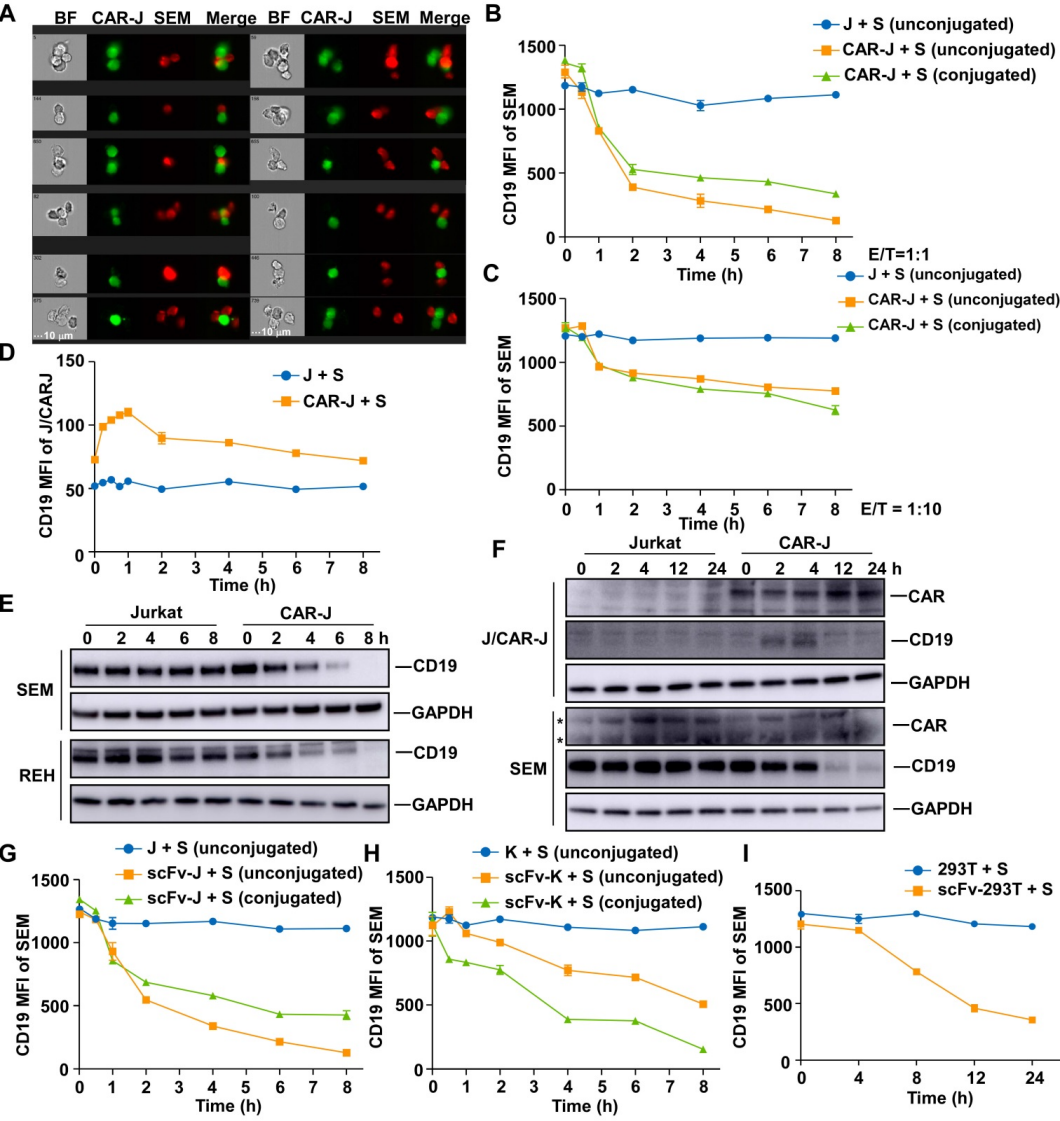
** Artificial antigen-recognizing cells cause antigen depletion on target cells.** (A) SEM cells were co-cultured with CD19-CAR-J cells for 1 h. Cells were monitored by image flow cytometry. BF, bright field. Scale bar, 10 μm. (B) MFI of CD19 in conjugated or unconjugated SEM cells after co-cultured with Jurkat or CAR-J cells. Conjugated and unconjugated cells were distinguished using FlowJo software. (C) MFI of CD19 in SEM cells co-cultured with Jurkat or CAR-J cells at the E:T ratio of 1:10. (D) MFI of CD19 in unconjugated Jurkat or CAR-J cells. (E) Immunoblots of target cells after co-culturing with Jurkat or CAR-J cells. (F) Immunoblots of SEM or Jurkat/CAR-J cells after co-culturing and sorting by flow cytometry. *, nonspecific bands. (G-I) MFI of CD19 in the indicated SEM cells co-cultured with CD19-scFv-J (G), CD19-scFv-K (H) or scFv293T (I) cells. Error bars reflect ± SEM.

Previous studies have found that CD19-CAR-T cells provoke reversible CD19 antigen loss through trogocytosis, an active process in which the target antigen is transferred to T cells 11. We first monitored the interaction between effector cells and target cells, and surmised that CD19-CAR-Jurkat T cells and SEM cells could form conjugates in various formats (Figure 2A and S2A). Since trogocytosis occurs at the immunological synapse and conjugate formation is a prerequisite 12, these conjugates could represent cells undergoing trogocytosis. When SEM cells were co-cultured with CD19-CAR-Jurkat T cells, the cell surface CD19 was remarkably reduced on the conjugated SEM cells (Figure 2B-C) and coincided with a slight CD19 increase on CD19-CAR-Jurkat T cells (Figure 2D), indicating that these cells did indeed undergo trogocytosis. The cell surface CD19 was also considerably reduced on the unconjugated SEM cells, even when co-cultured at a low E/T ratio of 1:10 (Figure 2B-C). We performed CD19 immunoblotting to confirm that the diminished CD19 antibody labeling resulted from CD19 depletion and not a competition between CAR molecules and CD19 antibodies 13. Figure 2E shows that CD19 protein levels were significantly reduced in B-ALL target cells when co-cultured with CD19-CAR-J effector cells. Notably, the increase in CD19 on effector cells was merely transient and not comparable to those lost on target cells (Figure 2B, D, F). These results indicated that the CD19 loss might not be principally through trogocytosis. To further exclude the possibility that these unconjugated SEM cells were actually those cells post-trogocytosis, SEM cells were co-cultured with non-signaling CD19-scFv-Jurkat T cells, which were incapable of mediating trogocytosis, since T-cell-mediated trogocytosis requires signaling of T cells 12. Interestingly, CD19 was consistently lowered on the target SEM cells (Figure 2G), and CD19 depletion was observed even when co-cultured with CD19-scFv expressing K562 (Figure 2H) or 293T cells (Figure 2I). These results implied that besides CAR-T cells, other artificial CD19-recognizing cells could also intrinsically cause cell surface CD19 depletion on the target cells through an unknown mechanism independent of lymphocyte-mediated trogocytosis.

We next examined whether the phenomenon of antigen depletion could extend to other cell types and antigens. Even though CD19-scFv-K562 cells could not kill RAJI cells, they could induce effective CD19 antigen depletion in RAJI cells (Figure S2B). Furthermore, CD22 and CD133 targeting effector cells could also induce effective antigen depletion in the corresponding target cells (Figure S2C-F). Thus, antigen depletion was a general feature of artificial antigen recognizing cells, which can probably be applied to many, if not all, antigens and cell types.

### CD19 depletion is mediated by endocytosis in target cells

**Figure 3:**
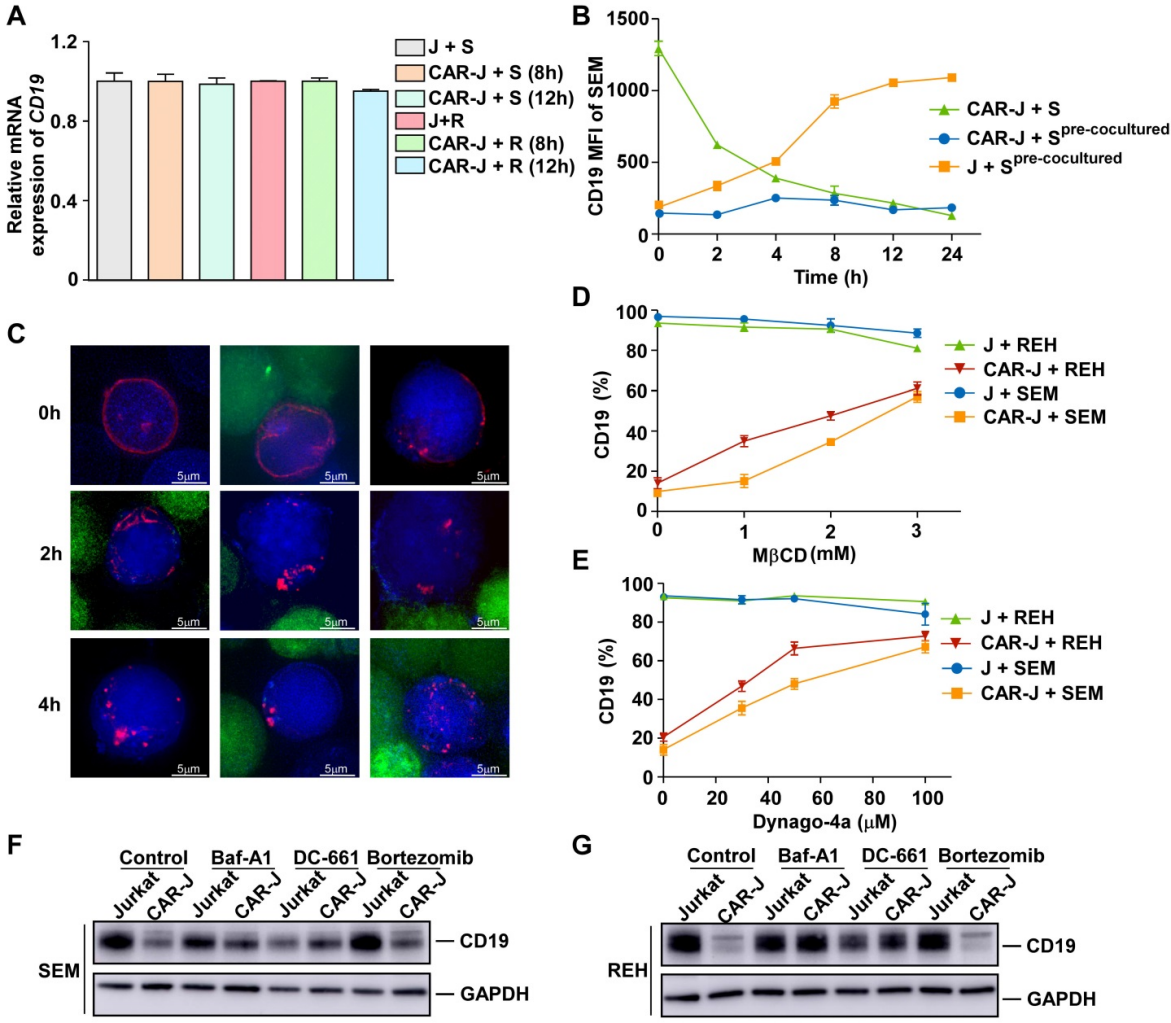
** CD19 depletion is mediated by endocytosis in target cells.** (A) SEM or REH cells were co-cultured with CAR-J or control Jurkat cells for the indicated times. The relative *CD19* mRNA levels in the target cells were evaluated by qRT-PCR. Values were normalized against* GAPDH*. (B) SEM cells were co-cultured with CAR-J cells for 8 h and then the target cells were sorted by flow cytometry. Fresh SEM cells or pre-cocultured SEM cells were re-cocultured with CAR-J or control Jurkat cells and CD19 expression in the target cells was analyzed by flow cytometry. (C) REH cells were co-cultured with CAR-J cells and the CD19 protein was monitored by DeltavVison OMX SR imaging system. The protease inhibitor cocktail was added to prevent protein degradation. Blue color indicates REH cells, red color denotes CD19-mRuby2 and green color indicates CAR-J cells. (D, E) REH or SEM cells were co-cultured with indicated effector cells exposed to MβCD (16 h) or dynago-4a (24 h). The percentage of CD19 positive cells was detected. (F, G) SEM or REH cells were pre-treated with Baf-A1 (100 nM), DC-661 (10 μM) or Velcade (10 nM) for 2 h, followed by co-culturing with Jurkat or CAR-J cells. CD19 levels in the SEM (F) or REH (G) cells were analyzed by immunoblotting. Error bars reflect ± SEM.

Although CD19 was depleted upon exposure to CD19-targeting effector cells, there was little variation in *CD19* mRNA expression (Figure 3A). Moreover, we found that diminished CD19 expression in the pre-co-cultured SEM cells was reversed without CD19-targeting effector cells (Figure 3B), indicating a reversible and post-transcriptional mechanism for CD19 depletion caused by the presence of artificial CD19-recognizing cells. Interestingly, the status of CD19 depletion in pre-co-cultured SEM cells was maintained in the presence of CD19-CAR-Jurkat effector cells (Figure 3B), even though they could no longer effectively activate these effector cells (Figure 1D), further supporting our view that CD19 depletion caused by the presence of artificial CD19-recognizing cells was T-cell activation independent.

We addressed the mechanism underlying CD19 depletion by first tracking the behavior of CD19 proteins in the co-culture system of REH and CD19-CAR-Jurkat cells. To this end, CD19-mRuby2 fusion proteins were ectopically expressed in REH cells (Figure S3A), and their degradation was prevented by the protease inhibitor cocktail. In control target cells, CD19 proteins were distributed evenly on the cell surface (Figure 3C). However, after co-culturing with effector cells for 2 h, CD19 in the target cells tended to coalesce to discrete cell surface areas (Figure 3C). Interestingly, CD19 proteins in both conjugated and unconjugated target cells showed a similar pattern, indicating that a transient interaction with effector cells was sufficient to cause CD19 protein translocation. After an additional 2 h, CD19 protein molecules translocated further into discrete foci inside the target cells (Figure 3C), suggesting that they were internalized into the target cells. We determined whether the internalization of CD19 was mediated by endocytosis by examining whether CD19 depletion on the target cell surface could be prevented by various endocytosis inhibitors including cholesterol-depleting methyl-β-cyclodextrin (MβCD) 14 and the dynamin inhibitor Dyngo-4a. Indeed, CD19 internalization and depletion could be rescued by these endocytosis inhibitors in a dose-dependent manner (Figure 3D-E), confirming that the internalization of CD19 was mainly mediated by endocytosis.

The CD19 depletion after co-culturing suggested that endocytosis of CD19 was ultimately terminated. One of the main degradation pathways for endocytosed proteins is lysosomal degradation 15. Therefore, to evaluate the pathway causing CD19 degradation after endocytosis, we examined the effects of various lysosomal and proteasomal inhibitors on CD19 depletion. We found that the lysosomal inhibitors bafilomycin A1 and DC-661 significantly abolished CD19 depletion caused by co-culturing with CD19-CAR-J cells, while the proteasome inhibitor bortezomib had little effect (Figure 3F-G). Collectively, our results suggested that the CD19 antigen was internalized and degraded by lysosomes inside the target cells in the presence of CD19-targeting effector cells.

### CD19 depletion undermines the survival of B-ALL target cells by disrupting the CD19/AKT/MYC axis

**Figure 4:**
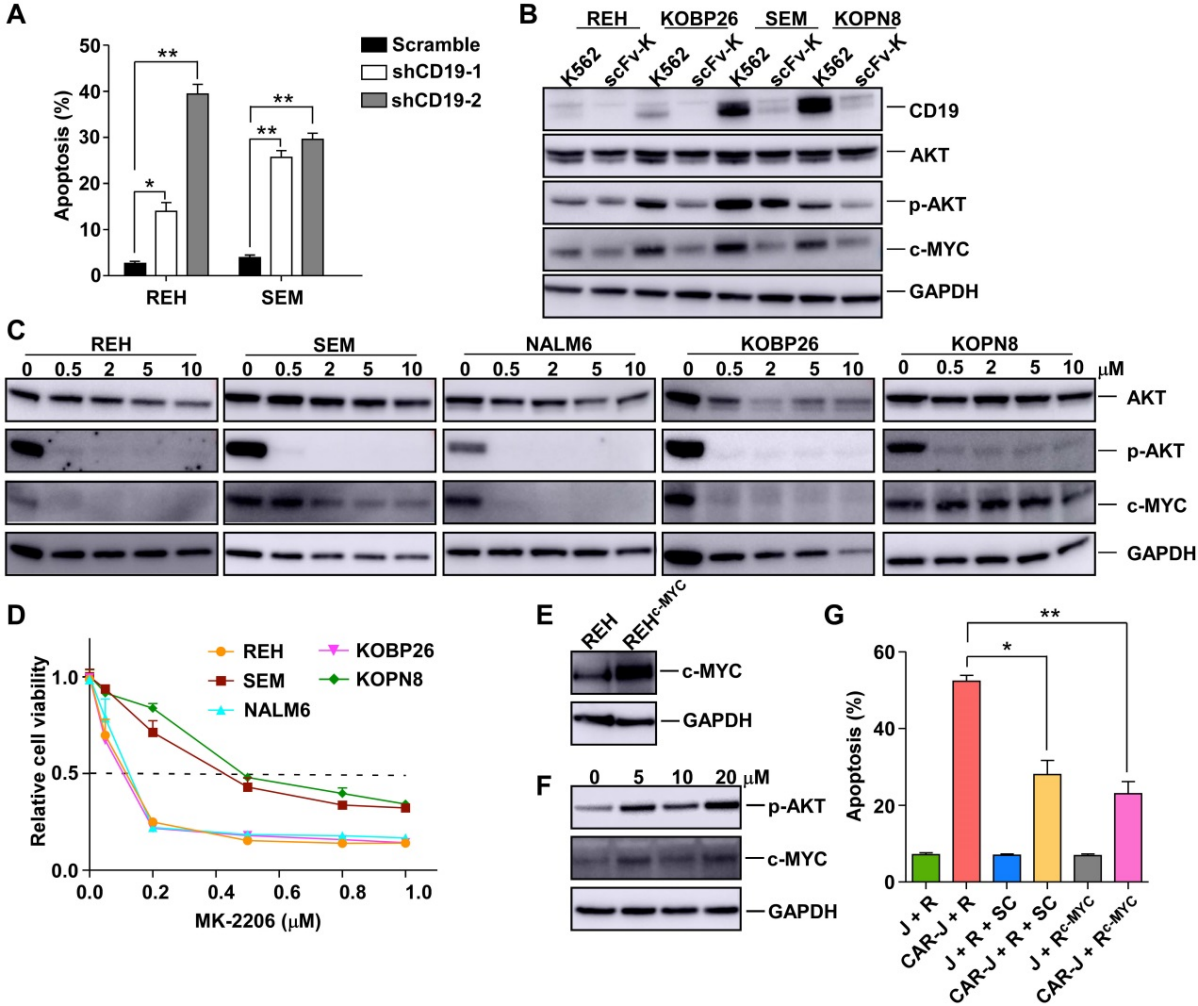
** CD19 depletion undermines the survival of B-ALL target cells via disruption of the CD19/AKT/MYC axis.** (A) Apoptosis analysis of REH or SEM cells infected with the indicated lentiviral vectors for 48 h. (B) Immunoblots of target cells co-cultured with CD19-scFv-K562 or control K562 cells. (C) Immunoblots of the indicated cells treated with the Akt inhibitor MK-2206 for 24 h. (D) Cell viability of the indicated cells treated with MK-2206 at the indicated concentrations for 5 days. (E) Immunoblots of REH cells infected with the indicated lentiviral vectors. (F) Immunoblots of the indicated cells treated with the Akt activator sc-79. (G) Jurkat or CAR-J cells were co-cultured with indicated target cells (R, REH; R+SC, REH cells treated with sc-79; R^c-Myc^, c-Myc overexpressed-REH cells). Apoptosis analysis of REH cells detected by flow cytometry. Statistical analysis was performed using the two-tailed t-test. Error bars reflect ± SEM.

As a cell surface signaling protein, CD19 is required for several processes involved in B-cell development and function, and is, therefore, essential for the survival of malignant B cells, and serves as a crucial therapeutic target for B-cell malignancies 16-18. Since the death of CD19 positive B-ALL cells caused by CD19-targeting AARC is accompanied by CD19 depletion in the target cells, we reasoned that CD19 depletion might account for the T-cell-activation independent chronic death effect of CD19-AARC. Indeed, acute CD19 depletion using CD19-targeting shRNAs led to apoptosis in REH and SEM cells (Figure 4A and S4A), suggesting that competent CD19 signaling was indispensable for the survival of these B-ALL cells, and CD19 depletion caused by CD19-AARC cells may lead to target cell death. In contrast, acute CD19 depletion could not kill RAJI cells (Figure S4B-C), suggesting competent CD19 signaling was dispensable for the survival of RAJI cells. These results explain why CD19-scFv bearing AARC cells could cause CD19 depletion but not cell death in RAJI cells.

The PI3K/AKT pathway is known to be instrumental in BCR signaling in B cells. High expression of CD19 on the malignant B cell surface may activate AKT and up-regulate MYC to support the survival of these cells 18, 19. MYC is one of the key oncoproteins in B cell malignancies, and its dysfunction appears to reduce cell proliferation and induce apoptosis uniformly 20. We determined whether the PI3K/AKT pathway and MYC expression in the target cells were compromised by CD19-AARC-mediated CD19 depletion. To this end, we examined the level of phosphor-AKT and MYC in the B-ALL target cells after co-culturing with CD19-scFv-K562 cells. As displayed in Figure 4B, decreased MYC levels were accompanied by the reduction of phosphor-AKT in CD19-depleted B-ALL cells. We treated the cells with the AKT inhibitor MK-2206 to determine whether the AKT/MYC axis dysfunction could cause cell death in B-ALL cells. With the deactivation of AKT, MYC protein levels were accordingly reduced in REH, NALM6, and KOBP26 cells and correlated with significantly reduced cell survival, confirming that MYC down-regulation could cause cell death (Figure 4C-D). Interestingly, in SEM and KOPN8 cells, whose survival was shown to be dependent on MYC 21, MYC protein levels were not accordingly reduced with AKT deactivation but were reduced only when a high concentration of MK-2206 was used. Thus, relative resistance to MK-2206 was observed in SEM and KOPN8 cells, implying that the CD19 depletion mediated down-regulation of MYC levels in these cells might also engage other unknown AKT-independent pathways that could be inhibited by the high concentration of MK-2206 (Figure 4B-D).

We further confirmed that MYC downregulation was essential for B-ALL target cell death by examining whether the reestablishment of MYC protein levels could rescue the target cell death. As expected, overexpression of MYC or rescuing the AKT/MYC axis using the AKT activator SC-79 in REH cells could mitigate the target cell death by CD19-scFv-K562 (Figure 4E-G). These results established that AARC-mediated CD19 depletion and MYC destruction caused target cell death mainly by disrupting the CD19/AKT/MYC axis.

### *In vivo* efficacy of CD19-AARC in a B-ALL xenograft mouse model

**Figure 5:**
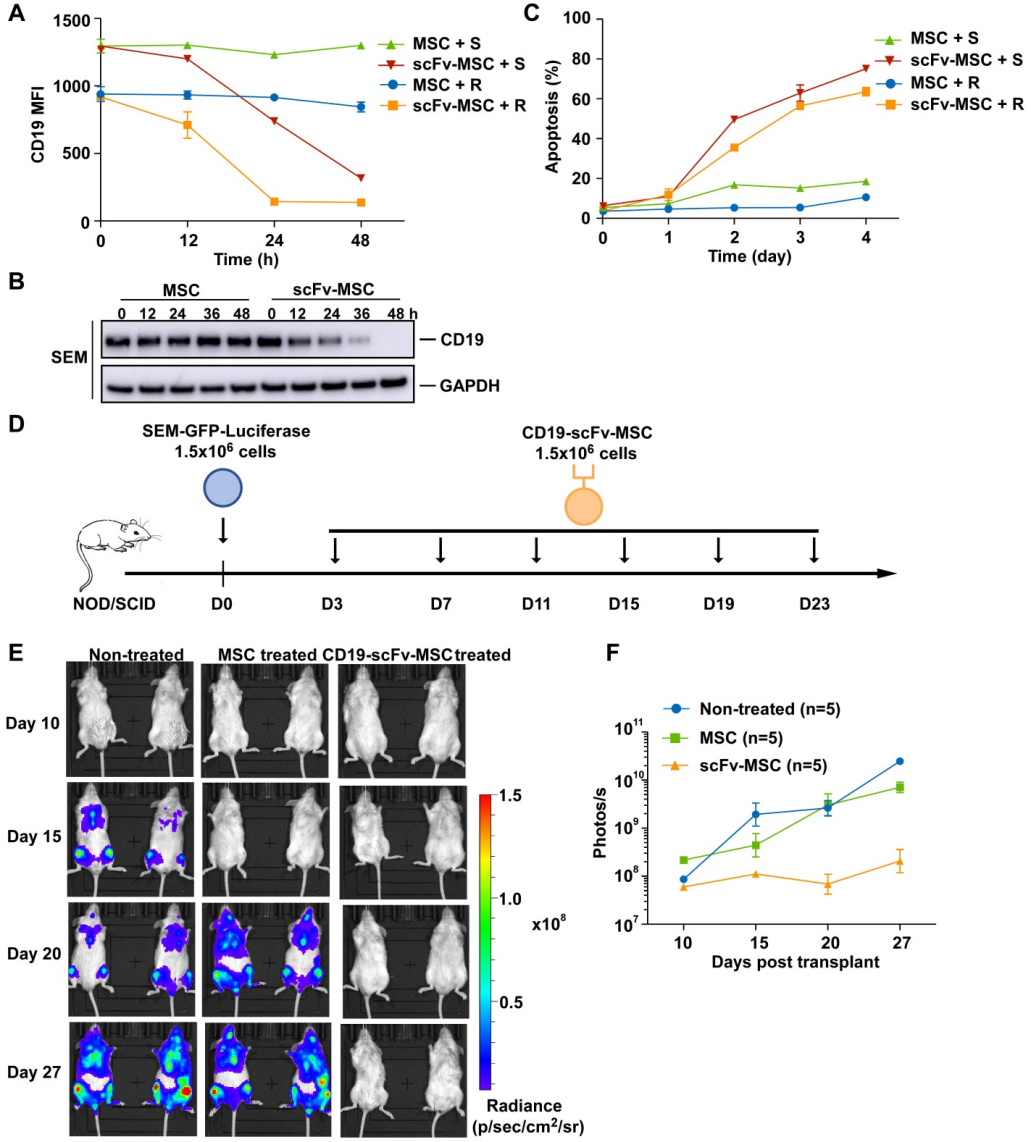
***In vivo* efficacy of CD19-AARC in a B-ALL xenograft mouse model.** (A) REH or SEM cells were co-cultured with CD19-scFv-transduced MSCs (CD19-scFv-MSC) or control MSC cells at the indicated times; CD19 levels in the target cells were determined by flow cytometry. (B) Immunoblots of SEM cells co-cultured with CD19-scFv-MSC or control MSC cells for indicated times. (C) Apoptosis of SEM or REH cells co-cultured with CD19-scFv-MSC cells determined by Annexin V staining. (D) Treatment schedule for CD19-scFv-MSC administration. (E, F) NOD/SCID mice (five in each group) transplanted with luciferase-expressing SEM cells and treated with CD19-scFv-MSCs. Mice were imaged at indicated days after xenografting to assess for leukemia progression. Representative bioluminescence images are shown on the left and the quantification of bioluminescence (photonic flux) over the duration of treatment is shown on the right. Error bars reflect ± SEM.

We next determined the *in vivo* efficacy of CD19-AARC cells in a previously established B-ALL xenograft mouse model 22. To this end, mesenchymal stromal cells (MSCs) were chosen as an AARC source because of their low immunogenicity, widely demonstrated clinical safety, good transplantability, and tumor-homing features 23. We examined the *in vitro* and *in vivo* efficacies of CD19-scFv presenting MSCs. Similar to other CD19-AARC cells, CD19-scFv-MSCs caused CD19 depletion and apoptosis efficiently in the co-cultured B-ALL target cells (Figure 5A-C). Subsequently, mice transplanted with SEM cells were treated with CD19-scFv-MSCs at the indicated interval and leukemia progression was monitored using bioluminescence (Figure 5D). Leukemic cells in the engrafted mice expanded rapidly in both untreated and control MSC treated conditions. In contrast, the engrafted mice treated with CD19-scFv-MSCs showed a striking reduction in leukemia progression (Figure 5E-F and S5A). Our results demonstrated both *in vitro* and *in vivo* efficacies of CD19-scFv-MSCs, suggesting the ability of CD19-AARC cells to serve as a novel cellular therapeutic approach for B-ALL leukemia treatment.

## Discussion

**Figure 6:**
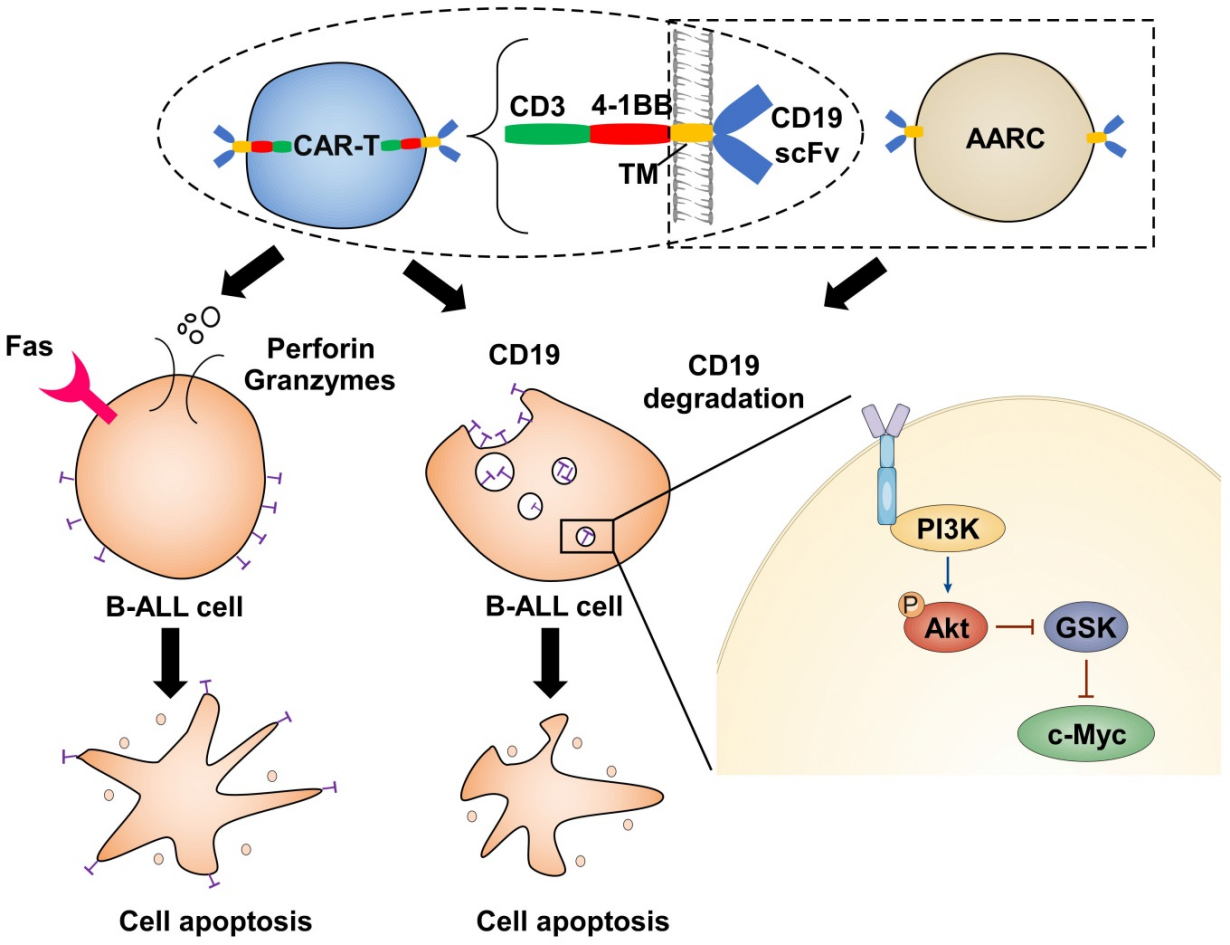
Illustration depicts how CAR-T cells induce CD19 degradation and CD19-dependent cell death.

Target cell death by CTLs is thought to be a rapid process. Therefore, a 4-hour cytotoxicity assay is routinely used to test the *in vitro* efficacy of CAR-T cells. However, the chronic effect of CAR-T cells has seldom been addressed. Here we have shown that the cytotoxic effect of CAR-T cells exerts both acute and chronic effects (Figure 6). The former is rapid and dependent upon T-cell activation, while the latter has much slower kinetics, is independent of T-cell activation, and relies on the depletion of the corresponding antigen. Importantly, only a fraction of the target cells may form stable associations with effector cells to initiate cell death through the classical acute CTL pathway. In contrast, most target cells can be deprived of the corresponding antigens through a transient interaction with effector cells and be destroyed through the chronic pathway. Thus, ideal CAR-T therapeutics with long-term efficacy should have a good chronic effect.

The chronic effect of CAR-T cells is largely dependent on antigen selection. In this context, our study provides valuable insights into antigen selection and CAR design. The first choice is that the antigen won't be internalized and depleted when recognized by the corresponding CAR. In this way, the escape of the antigen can be avoided and the target cells can be continuously recognized and destroyed. Alternatively, if the antigen is critical for the target cell survival, it can be internalized and depleted when recognized by effector cells. Thus, dysfunction of the antigen can lead to cell destruction.

Here we showed CD19 is a superior target because CD19-recognizing effector cells can maintain the target cells being deprived of cell-surface CD19, and therefore effectively cause chronic cell death in the target cells whose survival is dependent on CD19 signaling. This can partly explain why CD19-CAR-T remains the most successful CAR-T therapeutic. However, various antigens may have specific characteristics in different cancer cells, at least partly rationalizing the distinct response of cancers to different CAR-Ts or even the same CAR-T.

Consistent with previous reports 11, our results suggested that antigen escape is inevitable for some antigens, such as CD19. However, since the cells are dependent on these antigens, their escape can be manipulated into a cell death approach even though it may also prevent recognition of the cells by CAR-T cells. Although acute depletion of CD19 is detrimental to B-ALL cells, they can transform into an adaptive state, or develop clones with a growth advantage, so their survival is no longer dependent on CD19 16, 24. Based on our findings, the resistant cells might acquire the ability to survive in the absence of CD19. This might partly explain why different B cell malignancies have different relapse rates after CD19-CAR-T therapy 25.

The AARC could maintain the antigen depletion status of target cells through a transient interaction, which can endow them with cell death capability as a serial killer. Although antibody-based drugs, such as CD19 antibodies, can also cause antigen depletion 26 and kill or inhibit target cells by disrupting the antigen function 18, this effect is abolished since antibodies are co-internalized with their antigens. Thus, a sustainable antigen depletion status can be achieved by using excess antibodies or by constant infusion of antibodies to neutralize the recovery of the antigen. From this perspective, AARC, as a live drug, may have an advantage and transcend antibodies as a consumable drug.

The AARC can serve as the “off-the-shelf” ready-to-use therapeutic agent for large-scale clinical applications. The AARC treatment has a lower financial burden, no risk of manufacturing failure in certain patients or delay in treatment due to lengthy manufacturing procedures. Moreover, the non-lymphocytes based cell therapy is independent of T-cell activation, thus will greatly reduce the risk of CRS. Therefore, these universal cells are paving the way for a new generation of the allogeneic cellular therapeutic strategy being delivered to multiple recipients without re-editing of T cells.

For those who are not suitable to be treated with CD19 CAR-T, AARC treatment might provide an alternative option. In our study, MSCs were chosen as an AARC source because of the low immunogenicity, widely demonstrated clinical safety, good transplant potential, and tumor-homing features 23. Furthermore, MSCs have been administered in many clinical trials for multiple indications, making MSCs some of the most commonly used selected regenerative cells 27. The main design principle for generating AARC is to generate CD19-specific MSCs from allogeneic healthy donors. Due to easy isolation and high a proliferative rate, it is possible to obtain large amounts of MSCs for clinical use. In conclusion, CD19-MSCs may serve as an ideal complementary approach for CAR-T therapy.

## Supplementary Material

Supplementary figures.

## Abbreviations

CAR: chimeric antigen receptor
CRS: cytokine release syndrome
AARC: artificial antigen-recognizing cells
MSCs: mesenchymal stromal cells
scFv: single-chain antibody fragment
CTL: cytotoxic T lymphocytes
ACT: adoptive T-cell therapy

## References

[1] Depil S, Duchateau P, Grupp SA, Mufti G, Poirot L (2020). 'Off-the-shelf' allogeneic CAR T cells: development and challenges. Nat Rev Drug Discov.

[2] Martínez-Lostao L, Anel A, Pardo J (2015). How Do Cytotoxic Lymphocytes Kill Cancer Cells?. Clin Cancer Res.

[3] Benmebarek MR, Karches CH, Cadilha BL, Lesch S, Endres S, Kobold S (2019). Killing Mechanisms of Chimeric Antigen Receptor (CAR) T Cells. Int J Mol Sci.

[4] Turtle CJ, Hanafi LA, Berger C, Gooley TA, Cherian S, Hudecek M (2016). CD19 CAR-T cells of defined CD4+:CD8+ composition in adult B cell ALL patients. J Clin Invest.

[5] Hunder NN, Wallen H, Cao J, Hendricks DW, Reilly JZ, Rodmyre R (2008). Treatment of metastatic melanoma with autologous CD4+ T cells against NY-ESO-1. N Engl J Med.

[6] Liadi I, Singh H, Romain G, Rey-Villamizar N, Merouane A, Adolacion JR (2015). Individual Motile CD4(+) T Cells Can Participate in Efficient Multikilling through Conjugation to Multiple Tumor Cells. Cancer Immunol Res.

[7] Janssen EM, Lemmens EE, Wolfe T, Christen U, von Herrath MG, Schoenberger SP (2003). CD4+ T cells are required for secondary expansion and memory in CD8+ T lymphocytes. Nature.

[8] Braumüller H, Wieder T, Brenner E, Aßmann S, Hahn M, Alkhaled M (2013). T-helper-1-cell cytokines drive cancer into senescence. Nature.

[9] Xie Y, Akpinarli A, Maris C, Hipkiss EL, Lane M, Kwon EK (2010). Naive tumor-specific CD4(+) T cells differentiated *in vivo* eradicate established melanoma. J Exp Med.

[10] Smith MC, Luker KE, Garbow JR, Prior JL, Jackson E, Piwnica-Worms D (2004). CXCR4 regulates growth of both primary and metastatic breast cancer. Cancer Res.

[11] Hamieh M, Dobrin A, Cabriolu A, van der Stegen SJC, Giavridis T, Mansilla-Soto J (2019). CAR T cell trogocytosis and cooperative killing regulate tumour antigen escape. Nature.

[12] Aucher A, Magdeleine E, Joly E, Hudrisier D (2008). Capture of plasma membrane fragments from target cells by trogocytosis requires signaling in T cells but not in B cells. Blood.

[13] Ruella M, Xu J, Barrett DM, Fraietta JA, Reich TJ, Ambrose DE (2018). Induction of resistance to chimeric antigen receptor T cell therapy by transduction of a single leukemic B cell. Nat Med.

[14] Sanchez SA, Gunther G, Tricerri MA, Gratton E (2011). Methyl-β-cyclodextrins preferentially remove cholesterol from the liquid disordered phase in giant unilamellar vesicles. J Membr Biol.

[15] Settembre C, Fraldi A, Medina DL, Ballabio A (2013). Signals from the lysosome: a control centre for cellular clearance and energy metabolism. Nat Rev Mol Cell Biol.

[16] Sotillo E, Barrett DM, Black KL, Bagashev A, Oldridge D, Wu G (2015). Convergence of Acquired Mutations and Alternative Splicing of CD19 Enables Resistance to CART-19 Immunotherapy. Cancer Discov.

[17] Weiland J, Elder A, Forster V, Heidenreich O, Koschmieder S, Vormoor J (2015). CD19: A multifunctional immunological target molecule and its implications for Blineage acute lymphoblastic leukemia. Pediatr Blood Cancer.

[18] Chung EY, Psathas JN, Yu D, Li Y, Weiss MJ, Thomas-Tikhonenko A (2012). CD19 is a major B cell receptor-independent activator of MYC-driven B-lymphomagenesis. J Clin Invest.

[19] Katz BZ, Herishanu Y (2014). Therapeutic targeting of CD19 in hematological malignancies: past, present, future and beyond. Leuk Lymphoma.

[20] Dang CV (2012). MYC on the path to cancer. Cell.

[21] Zhu S, Cheng X, Wang R, Tan Y, Ge M, Li D (2020). Restoration of microRNA function impairs MYC-dependent maintenance of MLL leukemia. Leukemia.

[22] Liu H, Westergard TD, Cashen A, Piwnica-Worms DR, Kunkle L, Vij R (2014). Proteasome inhibitors evoke latent tumor suppression programs in pro-B MLL leukemias through MLL-AF4. Cancer Cell.

[23] Galipeau J, Sensébé L (2018). Mesenchymal Stromal Cells: Clinical Challenges and Therapeutic Opportunities. Cell Stem Cell.

[24] Weiland J, Pal D, Case M, Irving J, Ponthan F, Koschmieder S (2016). BCP-ALL blasts are not dependent on CD19 expression for leukaemic maintenance. Leukemia.

[25] Shah NN, Fry TJ (2019). Mechanisms of resistance to CAR T cell therapy. Nat Rev Clin Oncol.

[26] Sapra P, Allen TM (2002). Internalizing antibodies are necessary for improved therapeutic efficacy of antibody-targeted liposomal drugs. Cancer Res.

[27] Murray IR, Péault B (2015). Q&A: Mesenchymal stem cells - where do they come from and is it important?. BMC Biol.

