# Supplementary material for: A T-cell independent universal cellular therapy strategy through antigen depletion
Source: Theranostics. 2022 Jan 1;12(3):1148–60. doi: 10.7150/thno.66832 (PMC8771543; doi:10.7150/thno.66832)
Supplement: Supplementary file 1 — Supplementary figures. [file thnov12p1148s1.pdf]

Supporting Information

A T-cell independent universal cellular therapy strategy through antigen depletion

Dan Li[#], Wenbing Wang[#], Shufeng Xie[#], Maolin Ge[#], Ruiheng Wang, Qiongyu Xu, Yan Sun, Jiang Zhu, Han Liu*

Supplementary Figures

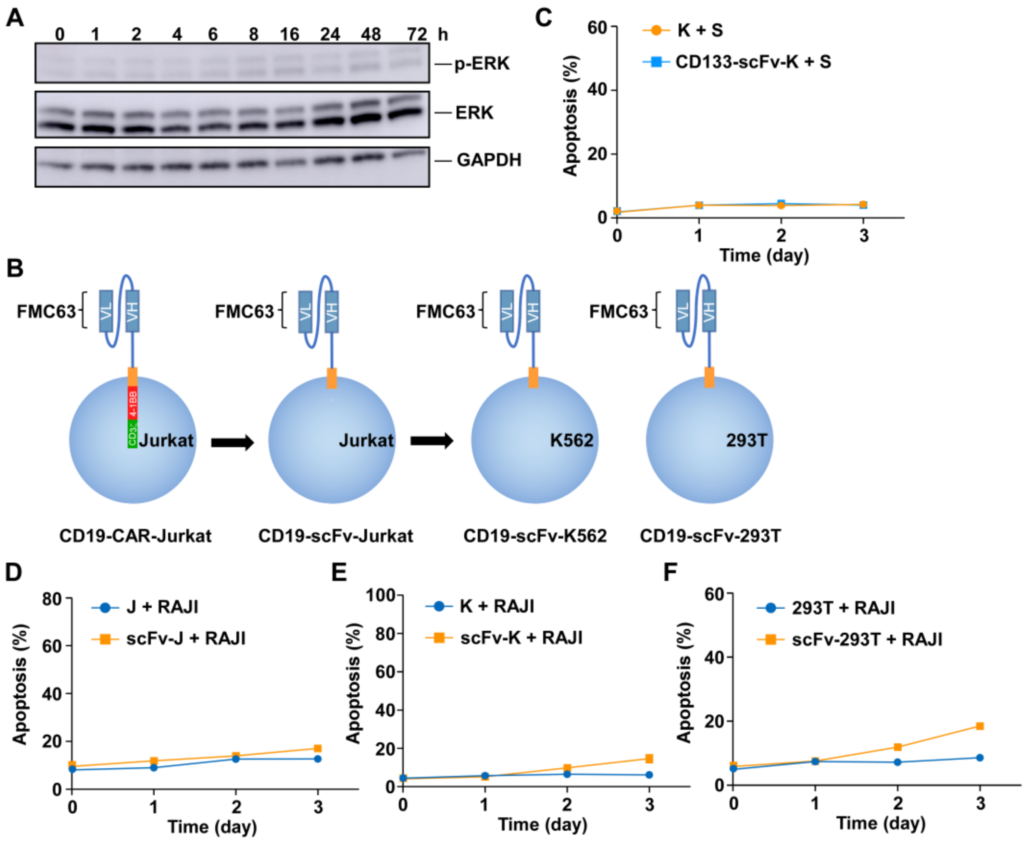

Figure S1 Target cell death through a T-cell independent mechanism. (A) The phosphorylated ERK levels of Jurkat T cells were determined after co-cultured with SEM cells for the indicated times. (B) The construction sketches of different effector cells. (C) Annexin V staining of SEM cells after co-cultured with CD133-scFv-K562 cells at the ratio of 1:1. (D-F) CD19 scFv-expressing Jurkat (scFv-J), CD19-scFv-K562 (scFv-K), or CD19-scFv-293T (scFv-293T) cells were co-cultured with CellTrace Far Red-labeled RAJI cells at the ratio of 1:1. The apoptosis of target cells was analyzed.

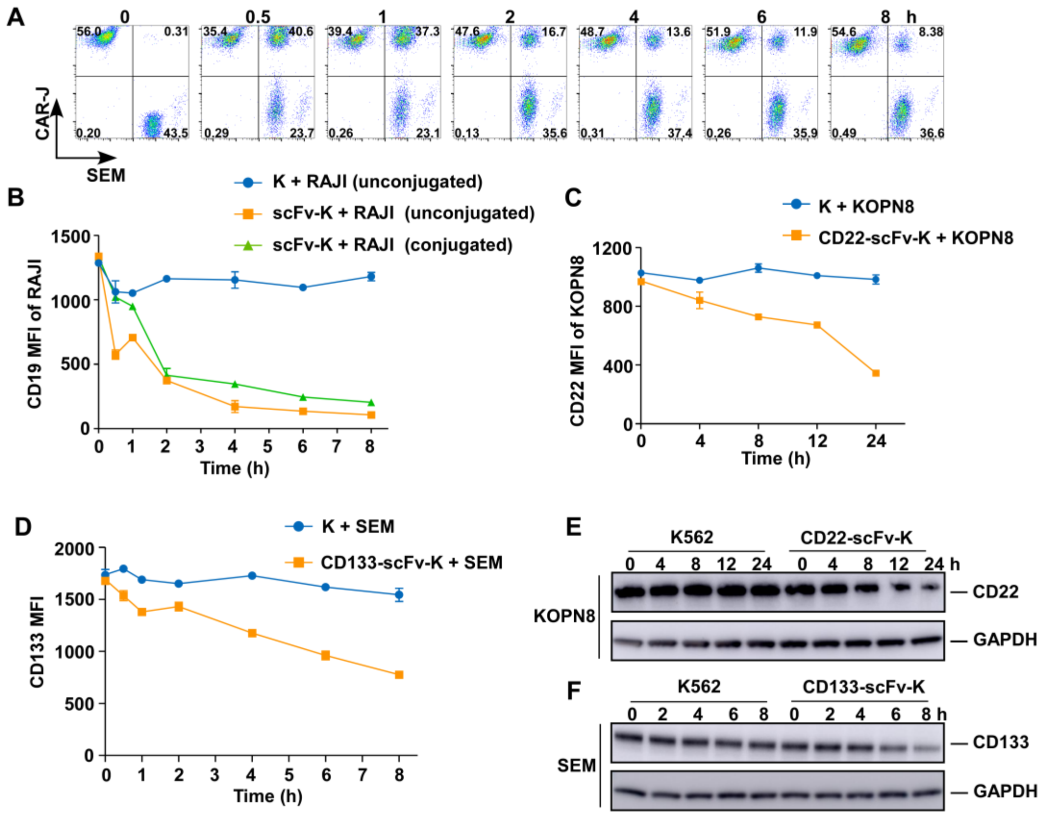

Figure S2 The phenomenon of antigen depletion on other antigens. (A) SEM cells were co-cultured with CD19-CAR-Jurkat T cells, and conjunction was detected at indicated times. (B) MFI of CD19 in RAJI cells was detected at indicated times. (C, D) CD22-scFv-K562 (CD22-scFv-K), or CD133-scFv-K562 (CD133-scFv-K) cells were co-cultured with the indicated target cells at the ratio of 1:1. The expression of CD22 and CD133 of target cells was analyzed. (E, F) Immunoblots of the indicated antibodies in target cells after co-cultured with the indicated effector cells.

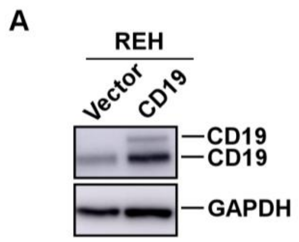

Figure S3 The overexpression of CD19. (A) The CD19 protein levels of REH cells infected by the indicated lentiviral vectors.

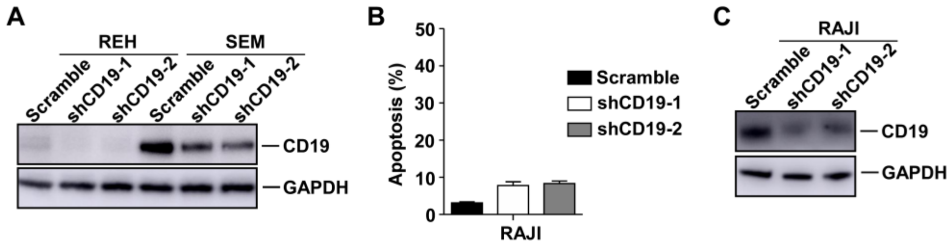

Figure S4 CD19 depletion in target cells. (A) The CD19 protein levels of REH and SEM cells infected by the indicated lentiviral vectors. (B) Apoptosis analysis of RAJI cells infected with the indicated lentiviral vectors for 48 h. (C) The CD19 protein levels of RAJI cells infected by the indicated lentiviral vectors.

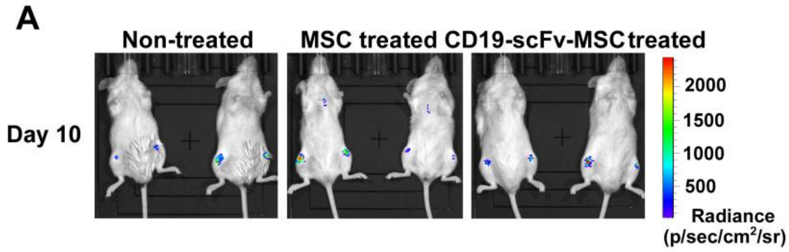

Figure S5 Bioluminescence imaging of B-ALL mouse model under the enhanced condition. (A) NOD/SCID mice were transplanted with luciferase-expressing SEM cells and treated with CD19-scFv-MSCs. Mice on day 10 after xenografting were imaged under enhanced model to assess for the successful inoculation.
